# Does the thermal evolution of molecular structures critically affect the magnetic anisotropy?†
†Electronic supplementary information (ESI) available. CCDC 1045631–1045633. For ESI and crystallographic data in CIF or other electronic format see DOI: 10.1039/c5sc01245g


**DOI:** 10.1039/c5sc01245g

**Published:** 2015-05-13

**Authors:** Kang Qian, José J. Baldoví, Shang-Da Jiang, Alejandro Gaita-Ariño, Yi-Quan Zhang, Jacob Overgaard, Bing-Wu Wang, Eugenio Coronado, Song Gao

**Affiliations:** a Beijing National Laboratory of Molecular Science , College of Chemistry and Molecular Engineering , State Key Laboratory of Rare Earth Materials Chemistry and Applications , Peking University , Beijing , 100871 , P. R. China . Email: gaosong@pku.edu.cn; b Instituto de Ciencia Molecular (ICMol) , Univ. de Valencia , C/Catedrático José Beltrán, 2 , E-46980 Paterna , Spain . Email: coronado@uv.es ; Email: gaita@uv.es; c LNCMI-CNRS , 25 rue des Martyrs BP 166 , 38042 Grenoble Cedex 9 , France . Email: jiang@lncmi.cnrs.fr; d Jiangsu Key Laboratory for NSLSCS , School of Physical Science and Technology , Nanjing Normal University , Nanjing 210023 , P. R. China; e Center for Materials Crystallography and Department of Chemistry , Aarhus University , Langelandsgade 140 , Aarhus DK-8000 , Denmark

## Abstract

A dysprosium based single-ion magnet is synthesized and characterized by the angular dependence of the single-crystal magnetic susceptibility. *Ab initio* and effective electrostatic analyses are performed using the molecular structures determined from single crystal X-ray diffraction at 20 K, 100 K and 300 K. Contrary to the common assumption, the results reveal that the structural thermal effects that may affect the energy level scheme and magnetic anisotropy below 100 K are negligible.

## Abstract 2

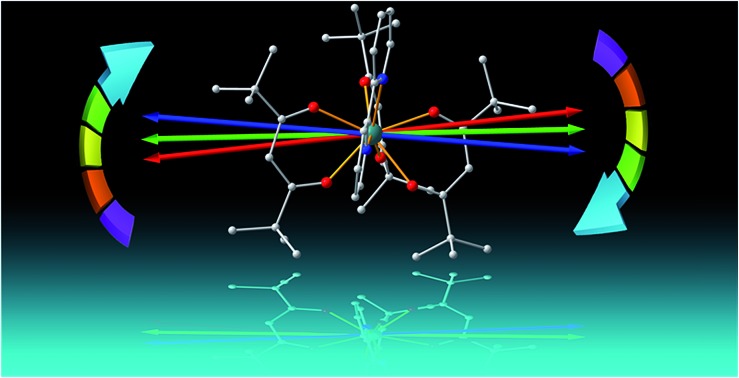
In the absence of a critical phase transition, one can safely use the crystal structure information determined at liquid nitrogen temperature in magnetic anisotropy research.

## Introduction

The magnetic properties of lanthanides have excited researchers for decades. The large anisotropy resulting from the unquenched orbital momentum and crystal field effects plays an important role in molecular magnetism. In particular, in the field of molecular magnetism, a seminal discovery was the obtainment of a 4f ion mononuclear complex showing single-molecule magnet (SMM) behaviour^1^ in 2003.^2^ Since then, the impact of these mononuclear SMMs, also known as single-ion magnets (SIMs), has dramatically increased.^3^–^6^ These kinds of coordination compounds are amongst the most complex magnetic entities with a large number of attractive physical properties such as slow relaxation of the magnetization and magnetic hysteresis at low temperatures, as well as quantum phenomena.^7^,^8^

In contrast to the classical cluster SMMs discovered in the 1990s,^1^ the properties of which are governed by exchange interactions, the magnetic properties of SIMs depend primarily on the single ion anisotropy resulting from spin–orbit coupling and the crystal field. The magnetic and spectroscopic properties of lanthanides can be fully understood by crystal field theory, which requires the determination of a large number of crystal field parameters (CFPs). This is a non-trivial task with only a few alternatives nowadays. The first one, broadly used by spectroscopists, is the extraction of phenomenological CFPs from a direct fit of the measured spectroscopic information. These parameters can thus be extracted from the optical, infrared or inelastic neutron scattering spectra. Experimental advances in the field aiming to address this issue include the high resolution luminescence spectroscopic method, which has been applied to determine the fine electronic structures of lanthanide complexes^9^,^10^ and, more recently, the work of Sessoli and coworkers using a single crystal torque magnetometry technique at various magnetic fields and a large range of temperatures.^11^ From the theoretical point of view, the CONDON program was developed by Lueken *et al.*, which uses the full Hamiltonian and determines the phenomenological CFPs from a fitting of the magnetic susceptibility data.^12^,^13^ Nevertheless, these kinds of approaches require an initial set of CFPs in order to avoid overparametrization and because of that, the substitution of the real structure by an ideal symmetry is of crucial importance. Attacking the problem from a different angle, there are several models that use the crystallographic structure to calculate CFPs. The simplest one is based on the point charge electrostatic model (PCEM),^14^ subsequently improved by several *semiempirical* models.^15^–^20^ A more expensive approach is to calculate the energy levels using *ab initio* methods. In general, the latter calculations have been the default option for the theoretical characterisation of SIMs. However, evidence of important deviations between the latter calculations and the experiments has been accumulating recently.^21^–^23^ In some studies, unphysical scaling factors have been employed to bridge this gap,^22^,^23^ which are attributed to either the thermal evolution of the molecular structures upon temperature variation or to the limitations of *ab initio* calculation capability.^13^

Nowadays, most of the experimental and theoretical investigations on the energy levels and magnetic anisotropy are performed based on the crystal structure determined at temperatures higher than that of liquid nitrogen, whereas the spectroscopic or the magnetic anisotropy experiments have been carried out at much lower temperatures. Therefore, a general question arises: can this thermal evolution of the structure critically affect the crystal field splittings and the magnetic anisotropy? In the present work, we aim to perform for the first time a detailed study of the molecular structure evolution effect at different temperatures and evaluate its consequences on the electronic and magnetic structure.

With this goal in mind, we report a dysprosium based β-diketonate SIM, Dy(^*t*^Bu-acac)_3_bpy (**1Dy**), where ^*t*^Bu-acac = 2,2,6,6-tetramethylheptane-3,5-dionate and bpy = bipyridine. Beyond the routine magnetic characterisation, the molecular magnetic easy axis was determined by angular resolved magnetization measurements on a single crystal. Based on this result, we are able to compare the precision of two different theoretical approaches, *i.e.* the *semiempirical* effective crystal field Hamiltonian approach, and *ab initio* calculations, where atom coordinates from single crystal X-ray crystallography at 20, 100 and 300 K were employed as inputs. The temperature effects upon the energy levels, CFPs and ground state wave functions are therefore elucidated.

## Experimental and calculation details

**1Dy** was synthesized as an amorphous powder and then purified by recrystallisation. An aqueous solution (3 mL) of KOH (3.0 mmol) was added to a heated ethanol solution (20 mL) of H^*t*^Bu-acac (3.0 mmol) and bpy (1.0 mmol) under stirring. A solution of Dy(NO_3_)_3_·6H_2_O (1 mmol) in 5 mL H_2_O was added dropwise to the above ethanol solution and the coarse products were obtained as a pale sediment. Suitable samples for structure determination and magnetic characterisation were recrystallized from a mixture of ethanol and acetone of the same volume ratio with a yield of 55.1%.

The X-ray diffraction data at 100 and 300 K were obtained using MoK_α_ radiation (*λ* = 0.71073 Å) with a graphite monochromator, while the 20 K data were collected on a synchrotron with liquid He for cooling.

The determination of the magnetic principal axes of low-symmetry systems was first developed by Gatteschi, Sessoli and their coworkers.^10^,^24^ This approach was soon proven to be very efficient and important in understanding the magneto-structure relation of rare earth ions.^9^,^25^,^26^ Herein, we applied a similar method to identify the magnetic easy axis of the present low-symmetry Dy^III^ complex. Taking advantage of the parallel orientation of the main magnetic axis in the crystal, we were able to determine the orientation of the magnetic easy axis. As the main difference compared with the method by Gatteschi and Sessoli, we mounted a single crystal of 3.07 mg with its (001) face on an L-shaped Cu/Be support (Fig. S2.1†), rather than a Teflon cube, so that we enabled the crystal to perform a rotation near the horizontal spin axis. This rotation was made around three orthogonal axes of the support in the temperature range of 1.8 to 15 K. The detailed experimental procedure can be found in the literature.^27^

Two well-established independent theoretical approaches, *ab initio* calculations^28^ and the *semiempirical* electrostatic method based on the Radial Effective Charge (REC) model,^20^ were carried out to rationalize the magnetic data for both the single crystal and the powder sample. For the *ab initio* approach, we performed post Hartree–Fock calculations based on the relativistic quantum chemistry method CASSCF/RASSI/SINGLE_ANISO implemented in MOLCAS 7.8 program package.^29^ These complete-active-space self-consistent field (CASSCF) calculations were performed on the single molecule fragments from the single crystal structure determined at 20, 100 and 300 K. The basis sets for all atoms are atomic natural orbitals from the MOLCAS ANO-RCC library: ANO-RCC-VTZP for the Dy^III^ ion; VTZ for close O and N; VDZ for distant atoms. The calculations employed the second order Douglas–Kroll–Hess Hamiltonian, where scalar relativistic contractions were taken into account in the basis set and the spin–orbit coupling was handled separately in the restricted active space state interaction (RASSI-SO) procedure. The active space includes all 9 f-electrons in 7 active orbitals. We calculated all the roots in the active space. We have mixed the maximum number of spin-free states which was possible with our hardware (all from 21 sextets; 128 from 224 quadruplets; 130 from 490 doublets). Basis sets and other variables were fixed to be identical for the three calculations, so that any differences in the output originate from structure variations.

For the REC calculations, we used the SIMPRE computational package,^30^,^31^ where we simultaneously fit the powder magnetic susceptibility data (2–300 K) and single-crystal easy axis susceptibility (2–15 K) with the same weight. Considering these different temperature ranges, crystal structures determined at different temperatures were used for powder data (100 K), and for the single crystal (20 K). For this fit, the radial displacement (*D*_r_) and effective charge (*Z*_i_) for the bipyridine ligand were taken from a previous study,^32^ so that only two free parameters are scanned, namely *D*_r_ and *Z*_i_ of the oxygen atoms from the β-diketonate ligand (Fig. S3.3–S3.5†), the best fitted values of the parameters were *D*_r_ = 0.57 Å and *Z*_i_ = 0.677.

## Results and discussion

### Structure

**Fig. 1:**
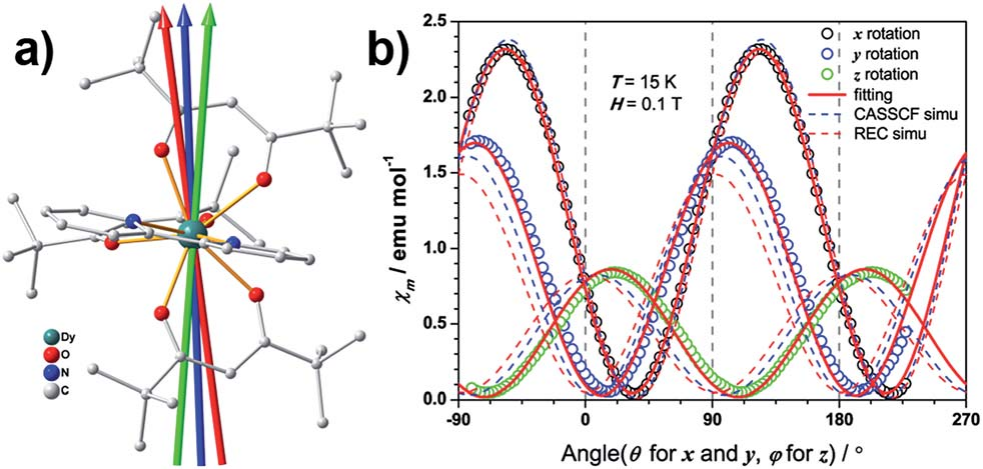
(a) View of the molecular structure of Dy(^*t*^Bu-acac)_3_bpy. The red, blue and green arrows indicate the magnetic easy axis directions determined from experiment, CASSCF calculation and the REC model, respectively. (b) Experimental (open circles) and simulated (curves) angular dependence of the magnetic susceptibility at 15 K. Solid and dashed curves represent the simulation from the best fitting of the experiment and the two theoretical results.

The X-ray single crystal structural analysis reveals that the molecule crystallizes in the triclinic *P*1 space group in the temperature range 20–300 K. The molecular structure as well as the unit cell changes slightly upon the variation of temperature. The crystal structures show that Dy^III^ is coordinated by six oxygen atoms from β-diketonate ligands, and two nitrogen atoms from bipyridine (Fig. 1a). As for many reported Dy/β-diketonate systems,^4^,^33^ the molecular structure can be viewed as forming a “paddle-wheel” shape, where the conjugated plane of each ligand forms the wheel. The anti-side wheels are nearly in the same plane, and the two planes from the four ligands are approximately perpendicular to each other. π–π stacking is found between bipyridine ligands of adjacent molecules at a separation of 3.3644 Å at 20 K. The two molecules in the unit cell are related by an inversion centre, indicating that the magnetic principal axes of these molecules are parallel to each other.

The Dy–O and Dy–N bond lengths at 20, 100 and 300 K are tabulated in Table 1. Although the unit cell shrinks upon cooling, the bond lengths at 20 K are not necessarily shorter than those at 300 K. The thermal variation is neither monotone nor trivial. As the first coordination sphere is not close to a perfect polyhedron, we decided to describe the symmetry in the lowest *C*_1_ point group. A Continuous Shape Measure analysis^34^ reveals that, taking the structure at 300 K as a reference, those at 20 K and 100 K are weakly distorted (*S* = 0.011 and *S* = 0.009, respectively) and almost identical to each other (each shows a *S* = 0.001 distortion taking the other one as a reference). Although irrelevant from a chemical point of view, these small thermal perturbations to the structure may have non-negligible effects on the magnetic properties.

**Table 1:** Bond lengths of Dy–N and Dy–O at 20, 100 and 300 K

Bond type	Dy–N1^*a*^	Dy–N2	Dy–O1	Dy–O2
20 K	2.5870(8)	2.5887(8)	2.3027(8)	2.3084(7)
100 K	2.582(3)	2.591(3)	2.297(2)	2.299(2)
300 K	2.589(4)	2.598(3)	2.307(3)	2.301(3)

Bond type	Dy–O3	Dy–O4	Dy–O5	Dy–O6
20 K	2.3539(8)	2.2821(7)	2.3283(8)	2.3425(8)
100 K	2.342(2)	2.281(2)	2.329(2)	2.328(2)
300 K	2.315(3)	2.281(3)	2.341(3)	2.329(3)

^*a*^The definitions of the atomic sequence numbers can be found in the ESI.†

### Magnetic measurements

**Fig. 2:**
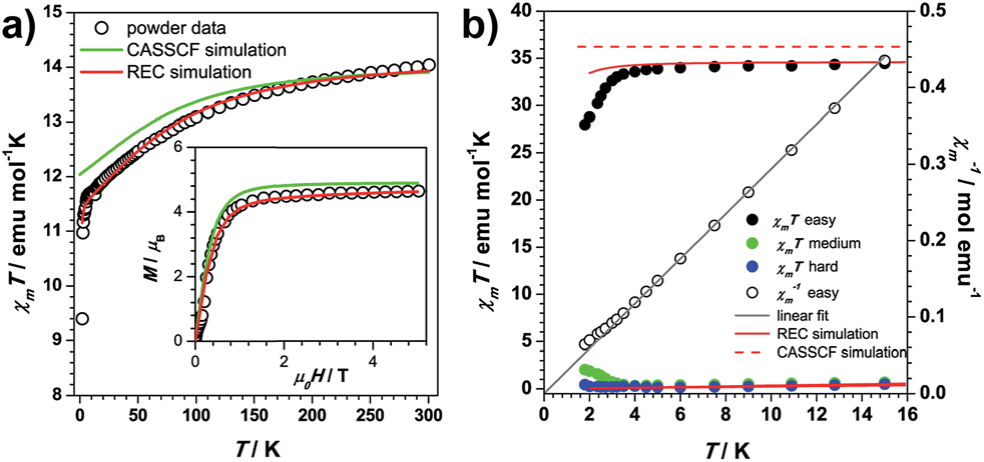
(a) Comparison of the *χ*_m_*T* value for powder from experiment, CASSCF and REC model simulation based on the structure at 20 K. Inset: comparison of the magnetization of powder at 2 K and theoretical prediction; (b) the *χ*_m_*T* along principal axes plotted against temperature.

The temperature dependence of the static magnetic susceptibility for **1Dy** shows a typical paramagnetic behaviour. The *χ*_m_*T* product gradually decreases upon cooling due to depopulation of the electronic fine structure and the antiferromagnetic dipolar interaction (Fig. 2a). A butterfly-shaped magnetic hysteresis is observed below 2 K and the hysteresis behaviour can be improved by magnetic site dilution (Fig. S1.1†). This kind of hysteresis is due to the slow spin lattice relaxation, however, before the single crystal data is recorded, one cannot directly relate this slow relaxation to an Ising type anisotropy.^35^,^36^

In the single crystal rotation, a sine curve was observed over the whole temperature range with a periodicity of 180° (Fig. S2.2†), but the sine curves below 3 K are not symmetric in all the three rotations. In a previous study it has been shown that the π periodicity can break down when slow magnetic relaxation exists.^37^ Therefore this deviation from the central symmetric behaviour can be attributed to the hysteresis effect when the system is suffering a non-equilibrium state of the magnetization. This occurs during the rotation below the blocking temperature. This can be verified from the splitting temperature of 3 K in the zero-field cooled and field cooled magnetization measurement taken under 1000 Oe, which is exactly the field employed in the single crystal rotation. The magnetic susceptibility tensor was obtained by a simultaneous fit of the rotation sine curves at the same temperature as the rotation functions (Fig. 1b). The magnetic easy axis orientation and the corresponding susceptibility value with respect to the experimental frame is determined after the diagonalization of the magnetic susceptibility tensor. The thermal variation of *χ*_m_*T* along the principal axes is plotted in Fig. 2b. Along the easy axis a constant value of 34 emu K mol^–1^ is observed, above 3 K, whereas along the other two directions the *χ*_m_*T* values are less than 0.5 emu mol^–1^ K. The direction of the easy axis is plotted in Fig. 1a. It is nearly in the plane of two β-diketonate ligands which are in the anti-side, as described before.

**Fig. 3:**
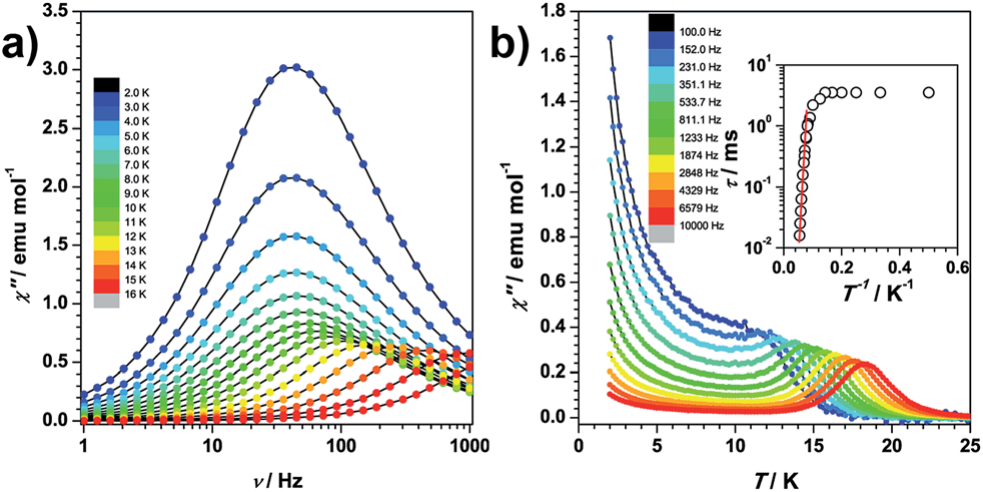
Out of phase dynamic susceptibility of the magnetically pure sample at zero field as a function of frequency (a) and temperature (b). Inset of (b): the relaxation time with respect to the inverse of temperature. The red line is the fitting to the Arrhenius law.

Dynamic magnetization of the magnetically pure and 5% diluted sample in the absence of an external field shows the presence of a frequency-dependent maximum in the out-of-phase signals (*χ*′′). For the undiluted sample, quantum tunnelling of magnetization is found at low temperatures, which is largely suppressed by dilution (Fig. 3 and S1.4†). The relaxation energy barrier at the higher temperature range is fitted to be 181 K in the pure analogue (Fig. S1.5†). To eliminate dipolar interactions, we illustrate the single ion behaviour on the diluted sample. In the absence of spectroscopic information, it is not wise to simply assume that the relaxation occurs *via* an Orbach process. We therefore independently fit the ac susceptibility data of the diluted sample to either an Orbach or a Raman relaxation process (Fig. S1.6†). The fitting of the relaxation time against temperature shows that a Raman process is unlikely since the Raman exponent of 12.7 is too large for Kramers systems with isolated doublets, while on the contrary the data were very well reproduced by the Arrhenius fit, indicating an Orbach process. Since the quantum tunnelling of magnetization process is also efficiently suppressed, it therefore makes sense to compare the theoretical energy gap with the experimental effective barrier *U*_eff_ = 189 K (131 cm^–1^).

### Theoretical analysis

Both *ab initio* and *semiempirical* calculations were conducted on the molecular structures determined at each of the three temperatures. Based on the 20 K structure, as shown in Fig. 2a, the CASSCF simulation deviates noticeably from the experimental data in the *χ*_m_*T* product below 200 K, while the information provided by the REC model is able to reproduce the magnetic susceptibility from both single crystal and powder samples over the whole temperature range, accurately predicting the field-dependent magnetization of the compound. In the latter case, the ground state wave function is found to be composed of 86% |±15/2> and 13% |±11/2>, with an effective spin of 1/2 having a *g*_//_ value of 19.11, very close to the value of 19.06 determined by the single crystal magnetization measurement (linear fit in Fig. 2b). In contrast, CASSCF calculations result in a near-Ising limit with *g*_//_ of 19.65. Additionally, the REC model is able to reproduce the single crystal magnetic susceptibility data for the easy, medium and hard axes between 2 and 15 K, while the CASSCF simulation of the easy axis behaviour is rather poor (Fig. 2b). On the other hand, both models produce very similar results in terms of the energy gap between the ground and first excited state within the ^6^H_15/2_ multiplet (Fig. 5a). Nevertheless, they do present important differences in the prediction of the higher energy levels in the multiplet (Fig. 5a), therefore further experiments are expected to verify the theory. In particular, in this case we are not able to perform single crystal magnetization measurements due to the largely reduced signal upon warming and the relative large background. The use of techniques such as cantilever torque magnetometry proposed by the group of Sessoli would be an interesting perspective to extend this work, due to its high sensitivity at higher temperatures.^11^

**Fig. 4:**
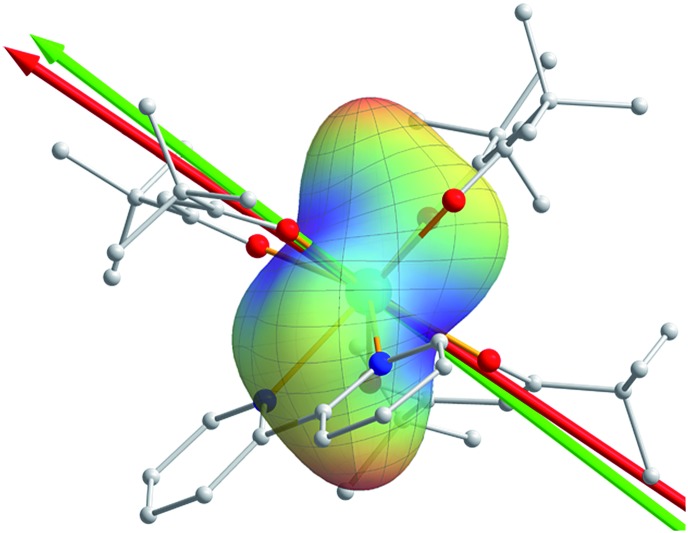
Magnetic principal axes, determined experimentally (red arrow) and calculated from the electrostatic potential (green arrow). As can be seen by the surface representation of the calculated relative potential energy, where blue is lowest and red is highest, the easy axis is oriented along the lowest potential energy direction.

**Fig. 5:**
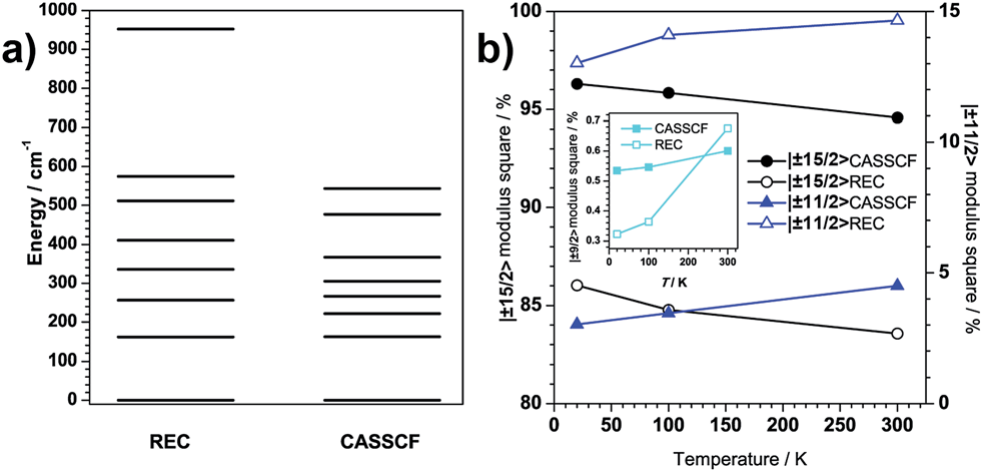
(a) Modulus square variation upon temperature of the dominant contributing wave functions (|±15/2> in circles, |±11/2> in triangles and |±9/2> in squares) to the ground state. (b) Energy levels calculated from REC model and CASSCF plot.

The magnetic easy axis predicted by both theoretical approaches are close to the experimental result at 4 K (with deviations of 5.9° and 12.3° for the CASSCF and the REC model, respectively). The calculated angular dependence of magnetic susceptibility at 15 K (Fig. 1b, dashed lines) agrees well with the rotation around the *x* axis, both in phase and magnitude. In contrast, obvious deviations can be found for the calculated susceptibilities in the *y* and *z* rotations. To gain an intuitive understanding of the orientation of the easy axis of magnetization, the electrostatic potential of the non-spherical 4f-electron cloud in the ground state interacting with its environment was calculated according to the idea proposed by Soncini *et al.*^38^ Since both the CASSCF and REC calculations result in a ground state wave function with a large weight of the *M*_J_ = ±15/2 base function, it is reasonable to approximate the anisotropic electron cloud to the Ising limit employing only an expansion of the axial spherical harmonics *Y*02, *Y*04 and *Y*06, thus reducing the complexity.^39^ The Mulliken charges of all the atoms were estimated from the CASSCF result. The direction of the easy axis is understood by recalling that the compressed aspherical electron cloud assumed by an Ising limit tends to orient the quantized axis along the direction of larger and closer negative charges. The potential energy landscape of Fig. 4 is due to the two anti-side β-diketonate ligands with four large negative charged oxygen atoms lowering the potential energy compared with the bpy and the last ^*t*^Bu-acac ligand, owing to the fact that the two coordinating nitrogen atoms bear relatively small charges and are further from the Dy^III^ ion. The present analysis is in agreement with previous work^35^,^37^ and the recently reported Dy/β-diketonate SIM.^26^ We would like to stress here that this simplified electrostatic approach does not rely on any fitting parameters and it is capable of providing the easy axis direction with a deviation of only 8.7° from the experimental one determined at 4 K. This deviation can be attributed to the aforementioned Ising limit approximation and to the neglected effect of the partially covalent character of the bond between the dysprosium and coordination atoms.

We now move on to evaluate the thermal effects of structural distortion on the electronic structures. Both theoretical approaches show that the contribution to the ground Kramers doublet wave function changes quantifiably with temperature (Fig. 5b). In particular, there is a downward trend in the relative weight of the *M*_J_ = ±15/2 contribution upon warming. It is necessary to note at this point that the present change of the quantum and magnetic properties is due to structural deformation that alters even the ground-state wave function, and not just because of the thermal population of excited wave functions. The evolution of the wave functions, though slight, could possibly affect the tunnelling process. Regarding the effect of the thermal distortion on the energy levels, both theories predict a slight decrease of the ligand-field splitting upon warming (Fig. S3.2†). It is interesting to realize that, at the same temperature, the CASSCF and REC models provide basically the same energy gap: 169 and 165 cm^–1^ at 20 K for REC and CASSCF, respectively; more shockingly, both methods predict a gap of 162 cm^–1^ at 100 K and a gap of 156 cm^–1^ at 300 K.

These results paint a picture where the most prominent feature is that, at least in the present Dy^III^/β-diketonate system, the thermal effects below 100 K on the magnetic properties are negligible. On one hand, the easy axis determined at 4 K forms an angle of 12.3°, 13.8° and 15.3° with the REC model based on the structure at 20, 100 and 300 K, respectively, while these angles for the CASSCF calculations are 5.9°, 6.2° and 7.9°. Both techniques demonstrate that the theoretically calculated magnetic axis does not seem to be sensitive to structural deformations owing to thermal effects. On the other hand, the main contribution to the ground state and the energy gap between the ground and first excited states remain within a 2% range. These results illustrate that the electronic and magnetic structures change upon the thermal evolution of the molecular structure within experimental error. We are therefore able to answer the title question: in the absence of a critical phase transition, one can safely use the crystal structure information determined at liquid nitrogen temperature in magnetic anisotropy research. The thermal effect of the molecular structure on the electronic structure does exist but it is negligible in practice.

It is interesting to compare the tiny thermal effect quantified here for the first time with the rather large scaling parameters that appear commonly in the literature and that are at least in part attributed to thermal effects. For example, our CASSCF calculations reveal a 2% deviation in the energy of the first excited level between 20 and 100 K, while scaling factors of up to 60% have been used to match low-temperature spectroscopy and state-of-the-art *ab initio* calculations^22^,^23^ using a crystal structure determined at 100 K.^13^ One can now conclude that these deviations are not due to thermal perturbations, but attributed to intrinsic methodological limitations of current *ab initio* methods, such as the necessarily limited size of the employed basis sets or unaccounted dynamical correlation. However, it is worth remarking that the failure in reproducing the magnetic data is at least partially due to the assumptions of the single ion model. In the present case and for many other SIMs, dipolar interactions between strong Ising anisotropic lanthanides rises abruptly by reducing the magnetic centre distance. One can expect that by introducing the effect of dipolar interactions in the CASSCF magnetic data simulation, part of the deviation from the experimental data could be corrected. Nevertheless, the dipolar interaction in the present case is too small to account for the whole difference between the experiment and calculation due to the large distance between Dy^III^ centers. Based on the CASSCF results, we evaluated the dipolar interactions for two types of molecular orientations in the lattice. The two molecules within the same unit cell form a sideways orientation of the easy axes with a distance of 12.3366 Å, whose coupling is calculated to be *J*_dip_ = 0.04 cm^–1^ (*JS*_1*z*_*S*_2*z*_ formalism). The pair of molecules in adjacent cells along the *b* direction form a head-on easy axes alignment separated by 12.2286 Å with *J*_dip_ = –0.08 cm^–1^. The couplings in both cases are very small compared to the crystal field effects and are not able to fill the gap between the experiment and calculation (Fig. S1.7†).

On the other hand, the REC model has been successfully applied here with a combination of both powder and single crystal magnetization data to reveal the magnetic anisotropy. One of its remarkable advantages compared to the CASSCF approach is its high efficiency. With the available molecular structure and magnetic data, one can, on a personal computer, rapidly interpret the important information associated with magnetic anisotropy, including the CFPs, crystal field splitting, wave function components, magnetic principal axes orientation and the magnetic susceptibility tensor at various temperatures.

Moreover, the obtained REC parameters for the ^*t*^Bu-acac ligand (*D*_r_ = 0.57 Å; *Z*_i_ = 0.677) have been used to perform a quick estimation of the energy level scheme and the temperature-dependent magnetic susceptibility of several Dy^III^ and Er^III^ β-diketonate related systems.^40^–^42^ As can be seen in Table S4.1,† the calculated energies of the first excited levels using CASSCF and the REC model are comparable. It is worth mentioning that the predicted results are pretty impressive, because we are assuming the same parametrization of the ligands of these related systems and it is obvious that they are not chemically identical to the ones investigated here. Furthermore, the *χ*_m_*T* product prediction is compared with the experiment (Fig. S4.1–S4.5†) and explains the SMM behavior of the five derivatives. The calculated easy axis orientation in these systems is represented in Fig. S4.6–S4.10.† As explained in previous studies,^43^,^44^ this strategy permits a rapid estimation of the magnetic properties in order to choose which metal would be more adequate to be surrounded by a concrete crystal field leading to SMM behaviour.

Of course, there are still some aspects that are necessary to discuss concerning this method if we aim to model all the observables of the system with high accuracy. The first point is that this model neglects the environment beyond the first coordination sphere and concerns only the coordinated atoms, or more precisely, the effective charges. This simplification can make the easy axis direction more sensitive to small perturbations in the coordination sphere, for example when molecular structures measured at different temperatures are used. The second limitation is that, for calculation simplicity, the current version of the SIMPRE code package^31^ is based on the Russell–Saunders coupling scheme, neglecting excited multiplets and inter-multiplet interactions. This approximation, despite its adequacy for heavy lanthanide ions, leads to small deviations in the predicted fine electronic structures, notably in the most excited levels,^31^ and, to a lesser extent, in the derived magnetic properties. This latter aspect can be improved using the SIMPRE calculated CFPs as an input in the CONDON package, which can refine the results using the full Hamiltonian, especially in systems with a lower number of CFPs or using the idealized symmetry. Last but not least, the *semiempirical* REC model is based on a single ion crystal field assumption, hence diluted samples are necessary in order to explain reliably the properties at low temperatures if dipolar interactions are not negligible in the system.

## Conclusions

This work, based on a combination of detailed experimental characterizations and two independent theoretical approaches, allows us to extract a few key insights. The most important one is that, for the first time, we have quantified the influence of the thermal evolution of the molecular structure in the electronic structure and magnetic anisotropy, and found it to be almost negligible, at least in the studied case of Dy^III^/β-diketonate, which possesses rather common features. In the absence of expected phase transitions, one can therefore trustingly employ the crystal structure determined at liquid nitrogen temperature to discuss the magnetic anisotropy properties, since the thermal effects below 100 K do exist but are negligible in practice. Regarding the energy level scheme, this means that one can also exclude that these small structural variations might be the source of the rather large scaling factors in the CASSCF calculations for crystal field splitting, as is our main second conclusion. In other words, the deviation of the *ab initio* calculation from the experiment should probably be attributed to the insufficient triple-zeta basis set that is commonly used or to fundamental limitations regarding dynamical correlations, not to structural effects. The studied complex does not have any extraordinary or even unusual chemical features, and this suggests that the conclusions we extract are of general utility. Nevertheless, it is worth noting that the extension of the present study in combination with spectroscopic as well as torque experiments to other systems and to intermediate temperatures is necessary before one can categorically discard non-negligible effects of thermal structural evolution on magnetic anisotropy as a general rule.

## Supplementary Material

Supplementary information

Crystal structure data
