# Supplementary material for: Does the thermal evolution of molecular structures critically affect the magnetic anisotropy?
Source: Chem Sci. 2015 May 13;6(8):4587–93. doi: 10.1039/c5sc01245g (PMC5851079; doi:10.1039/c5sc01245g)
Supplement: Supplementary file 1 [file SC-006-C5SC01245G-s001.pdf]

Electronic Supplementary Material (ESI) for Chemical Science.
This journal is © The Royal Society of Chemistry 2015

Supporting Information

Does the Thermal Evolution of Molecular Structures Critically Affect Electronic and Magnetic Structure?

List of Supporting Information

SI1 Magnetic measurements on powder sample

SI2 Single crystal measurements detail

SI3 Summary of the theoretical results

SI4 Prediction of the properties of five related β-diketonate complexes

SI1 Magnetic measurements on powder sample

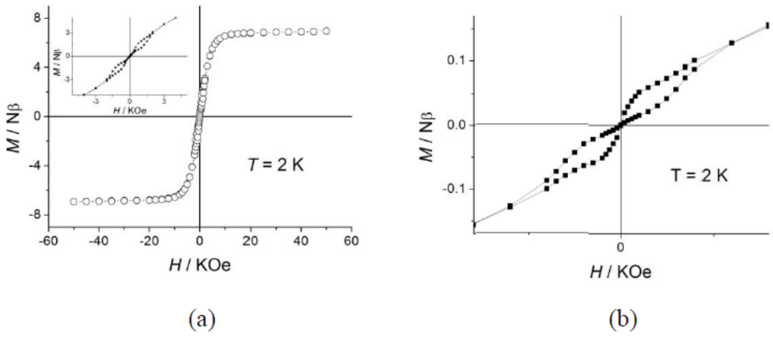

Fig. S1.1 The magnetic hysteresis measurement of 1Dy for pure sample (a) and magnetic site diluted sample (b). The dilution was made by Dy:Y mole ratio of 1:20.

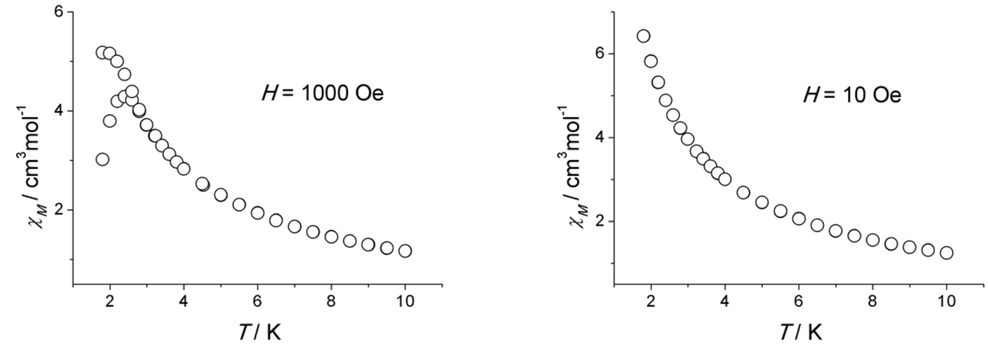

Fig. S1.2 Zero field cooled and field cooled magnetic susceptibility measurements at 1000 and 10 Oe.

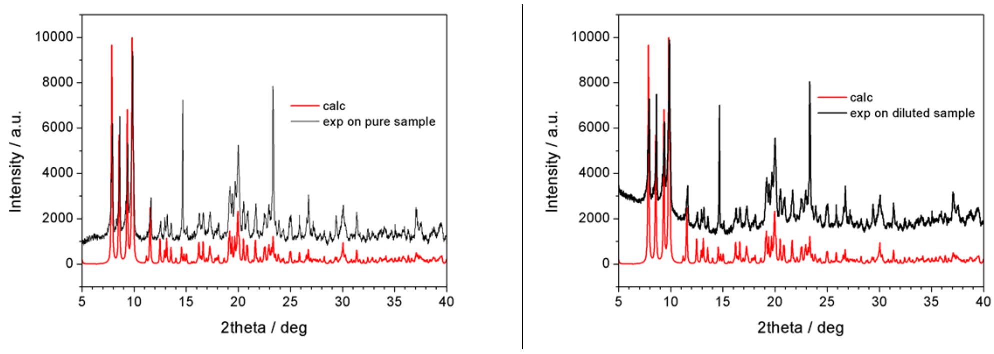

Fig. S1.3 the powder XRD data of pure and magnetic diluted sample compared to the simulation from the single crystal XRD data.

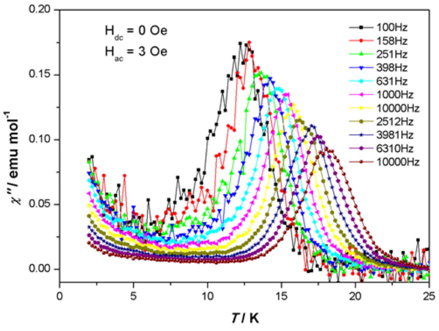

Fig. S1.4 the powder dynamic susceptibility data on powder sample in the absence of dc field.

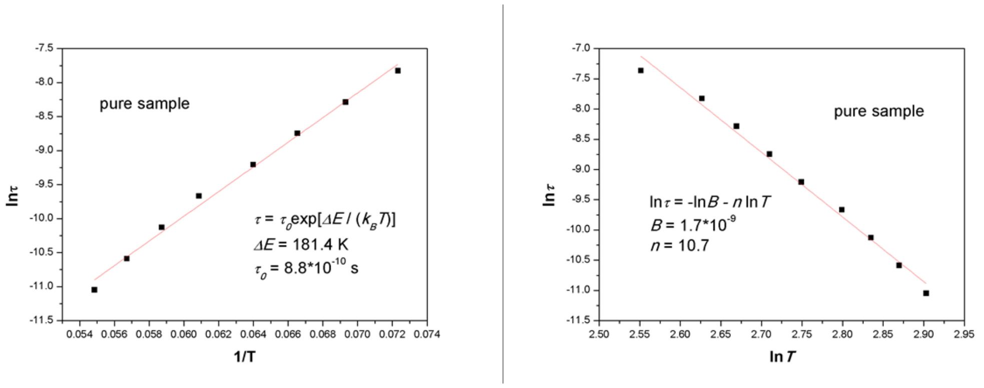

Fig. S 1.5 The relaxation time as a function of the temperature can be fitted to Orbach (left) and Raman (right) process on pure sample. The Orbach fitting results in a good linear relation of lnτ and 1/T. The Ramann fitting results in a slope of 10.7, far from the expected value of 9 for Kramers ion with isolated doublets.

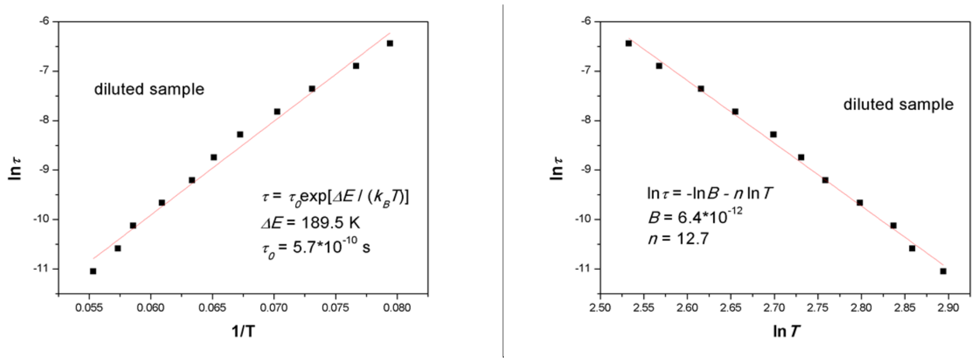

Fig. S1.6 The relaxation time as a function of the temperature can be fitted to Orbach (left) and Raman (right) process on magnetic diluted sample. The Orbach fitting results in a good linear relation of lnτ and 1/T. The Ramann fitting results in a slope of 12.7, far from the expected value of 9 for Kramers ion with isolated doublets.

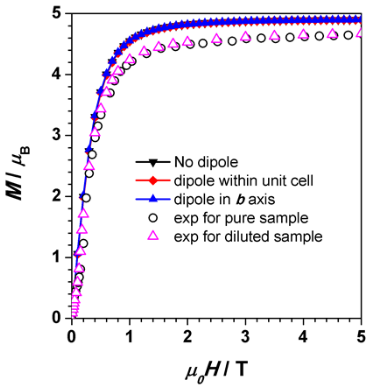

Fig. S1.7 The M v.s. H plot for experiment and simulation. The open triangle and circles are experimental data from 20 times magnetic site dilution and pure sample. The closed dots are based on the CASSCF results with no dipole (black), dipole interaction between easy axes in shoulder-on-shoulder orientation (red) and head-on orientation (blue). The differences of the three simulations are too small to observe, and neither can account for the deviation from the experiment result.

SI2 Single crystal measurements detail

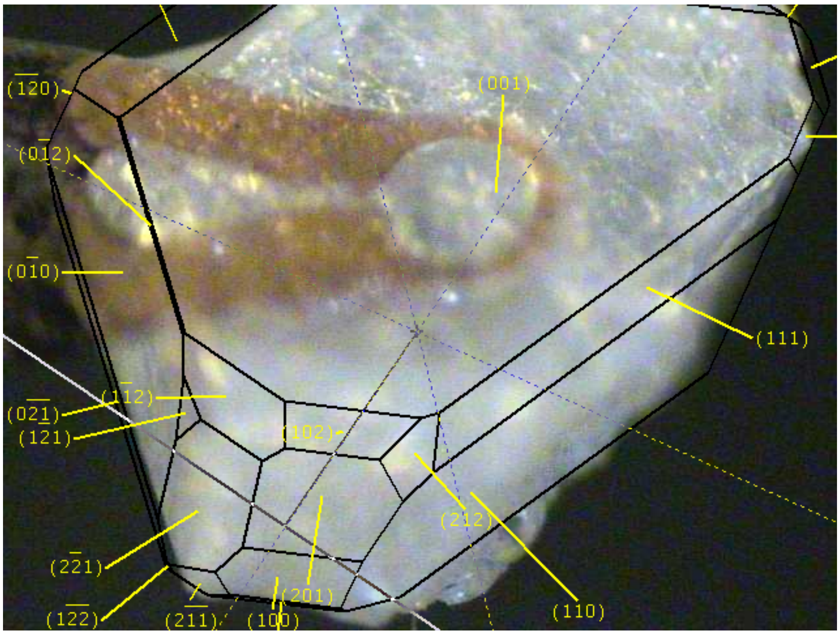

Fig. S2.1 The faceindex of the crystal.

(001) is defined as the XY plane

a axis (intersection of (001) and (010)) is the X axis

the transformation matrix between XYZ and ***abc*** is

$$\begin{pmatrix} a \\ b \\ c \end{pmatrix} = \begin{pmatrix} 11.0343 & 0 & 0 \\ 4.24374 & 11.4686 & 0 \\ 1.31436 & 3.68653 & 17.0915 \end{pmatrix} \begin{pmatrix} X \\ Y \\ Z \end{pmatrix}$$

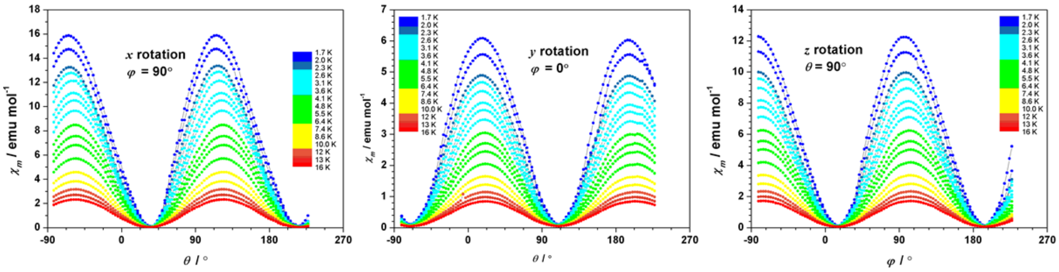

Fig. S2.2 The magnetic susceptibility along xyz directions at various temperatures.

Table S2.1 The list of magnetic easy axis and the relative angles (in degree)

	T (K)	normalized easy axis vector[a]			angle (deg) to					
					REC			CASSCF		
		X	***Y***	***Z***	20 K	100 K	300 K	20 K	100 K	300 K
Exp.	1.80	–0.16138	–0.84199	0.51480	11.7	13.2	14.4	5.7	6.0	7.1
	2.00	–0.17270	–0.83795	0.51770	12.2	13.7	14.9	6.1	6.4	7.6
	2.35	–0.16559	–0.83597	0.52320	11.7	13.2	14.4	5.5	5.9	7.1
	2.50	–0.16359	–0.83425	0.52656	11.5	13.0	14.3	5.3	5.6	6.9
	2.70	–0.16567	–0.82899	0.53416	11.4	12.9	14.2	5.1	5.5	6.8
	3.00	–0.17319	–0.82223	0.54216	11.7	13.2	14.5	5.3	5.6	7.1
	3.20	–0.17734	–0.81976	0.54456	11.9	13.3	14.7	5.4	5.7	7.4
	3.50	–0.18188	–0.81726	0.54682	12.1	13.6	15.0	5.6	5.9	7.6
	4.00	–0.18733	–0.81381	0.55011	12.3	13.8	15.3	5.9	6.2	7.9
	4.50	–0.19623	–0.80977	0.55296	12.8	14.3	15.7	6.3	6.6	8.4
	5.00	–0.19780	–0.80986	0.55227	12.9	14.4	15.8	6.4	6.7	8.5
	6.00	–0.19818	–0.80989	0.55209	12.9	14.4	15.9	6.5	6.8	8.5
	7.50	–0.19895	–0.80994	0.55175	13.0	14.4	15.9	6.5	6.8	8.5
	9.00	–0.19990	–0.81016	0.55108	13.0	14.5	16.0	6.6	6.9	8.6
	10.90	–0.19984	–0.81035	0.55082	13.0	14.5	16.0	6.6	6.9	8.6
	12.80	–0.19974	–0.81084	0.55013	13.0	14.5	16.0	6.6	6.9	8.6
	15.00	–0.19876	–0.81136	0.54972	13.0	14.4	15.9	6.5	6.8	8.5
REC	20	0.02227	–0.80310	0.59542	0	1.5	3.1	6.4	6.2	4.6
	100	0.04774	–0.79982	0.59834	1.5	0	1.8	7.9	7.6	6.1
	300	0.07550	–0.80611	0.58694	3.1	1.8	0	9.4	9.1	7.4
CASSCF	20	–0.08817	–0.81256	0.57618	6.4	7.9	9.4	0	0.3	2.2
	100	–0.08351	–0.81119	0.57879	6.2	7.6	9.1	0.3	0	2.1
	300	–0.05117	–0.82200	0.56718	4.6	6.1	7.4	2.2	2.1	0

[a] The xyz Cartesian system is defined in Fig. S2.1

SI3 Summary of the theoretical results

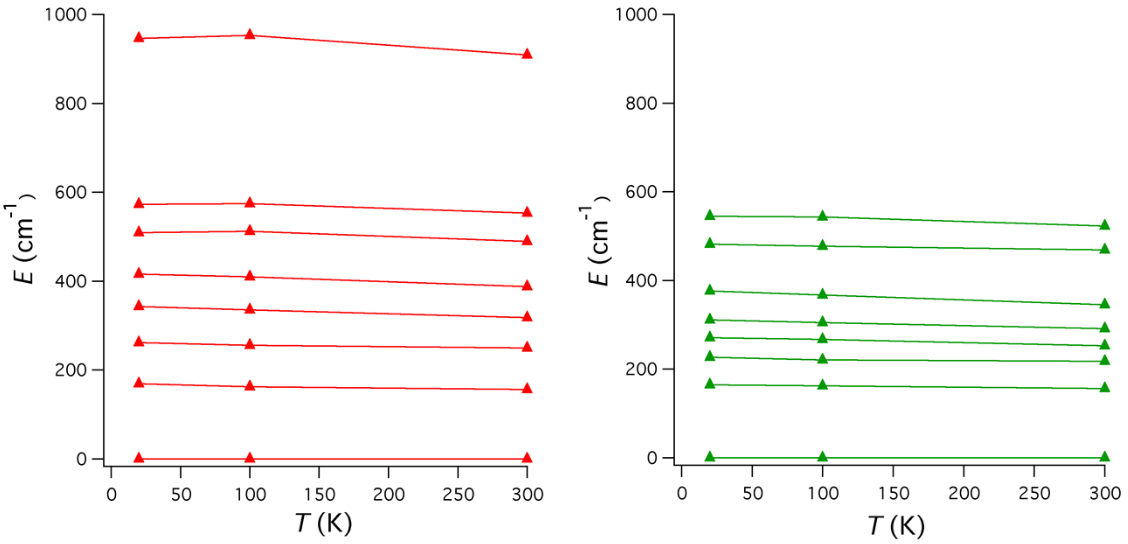

Fig. S3.1 Calculated lower lying magnetic energy levels using the crystallographic structures measured at 20 K, 100 K and 300 K; (left) REC model, (right) *ab initio*.

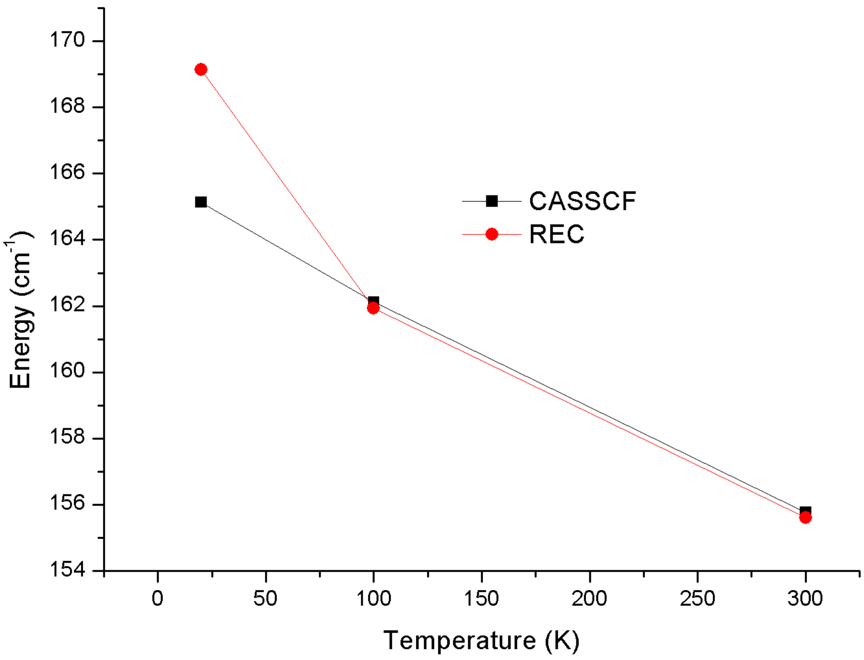

Fig. S3.2 Calculated energy of the first excited doublet using the crystallographic structures measured at 20 K, 100 K and 300 K as inputs (the ground doublet is shifted to 0).

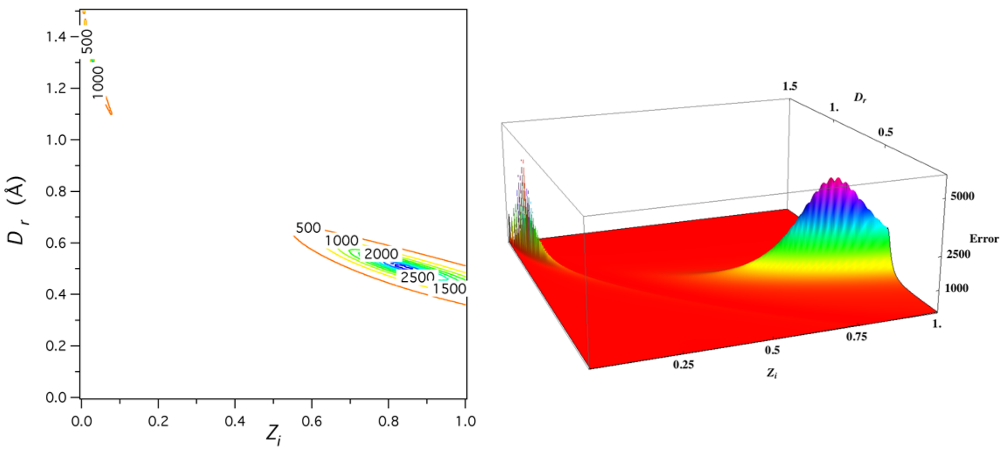

Fig. S3.3: 2D (left) and 3D (right) representation of the inverse of the relative error in the REC fitting of the powder χT product versus D_r (from 0 to 1.5 Å) and Z_i (from 0 to 1).

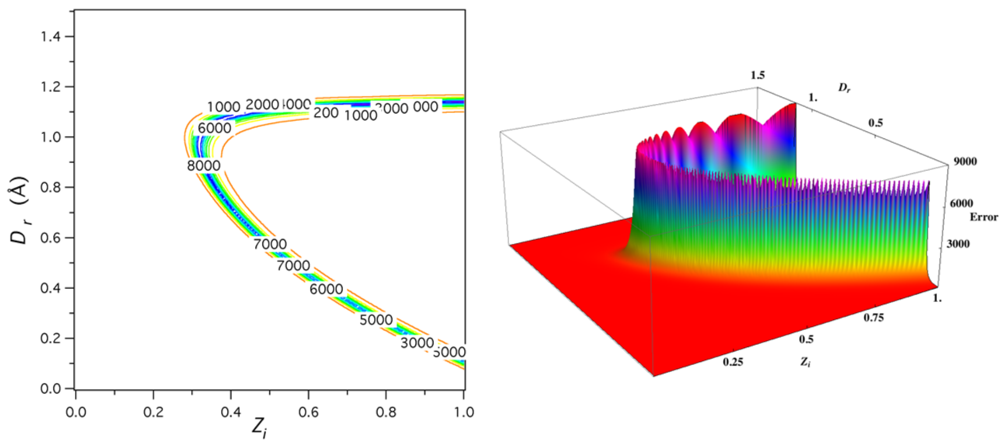

Fig. S3.4: 2D (left) and 3D (right) representation of the inverse of the relative error in the REC fitting of the single crystal easy axis χT product versus D_r (from 0 to 1.5 Å) and Z_i (from 0 to 1).

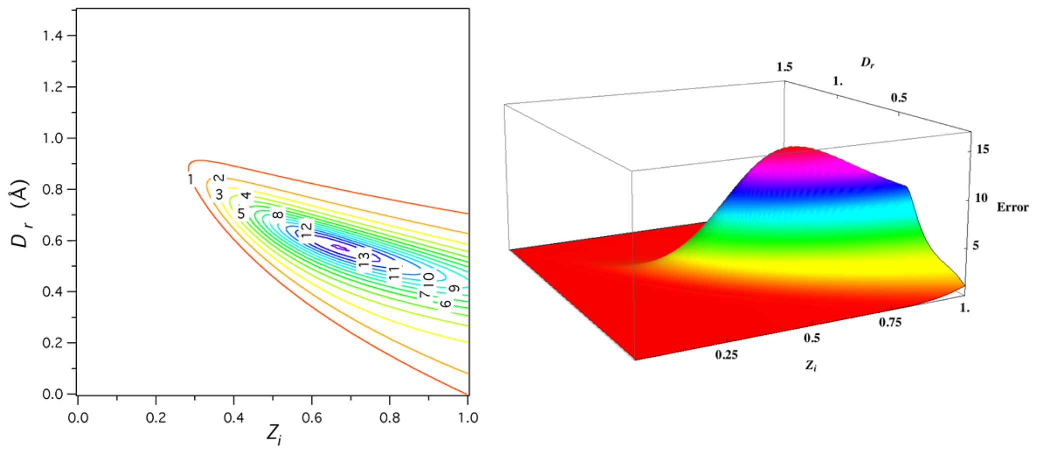

Fig. S3.5: 2D (left) and 3D (right) representation of the inverse of the average of the relative errors in the fitting of the powder and the single crystal easy axis χT product versus D_r (from 0 to 1.5 Å) and Z_i (from 0 to 1). The minimum error correspond to $D_r = 0.57$ Å and $Z_i = 0.677$.

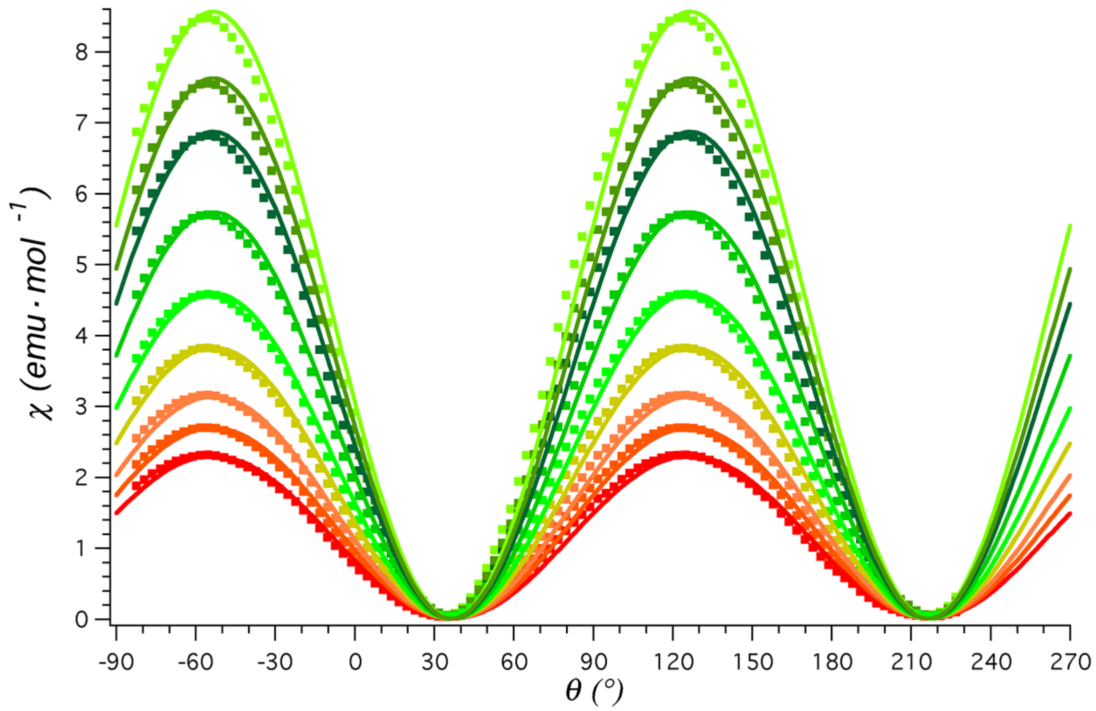

Fig. S3.6: Angular dependence of the magnetic susceptibility at different temperatures for Rot X at H = 1 kOe. From top to bottom: 4, 4.5, 5, 6, 7.5, 9, 11, 12.8 and 15 K. Experiment (solid circles); REC model (solid lines).

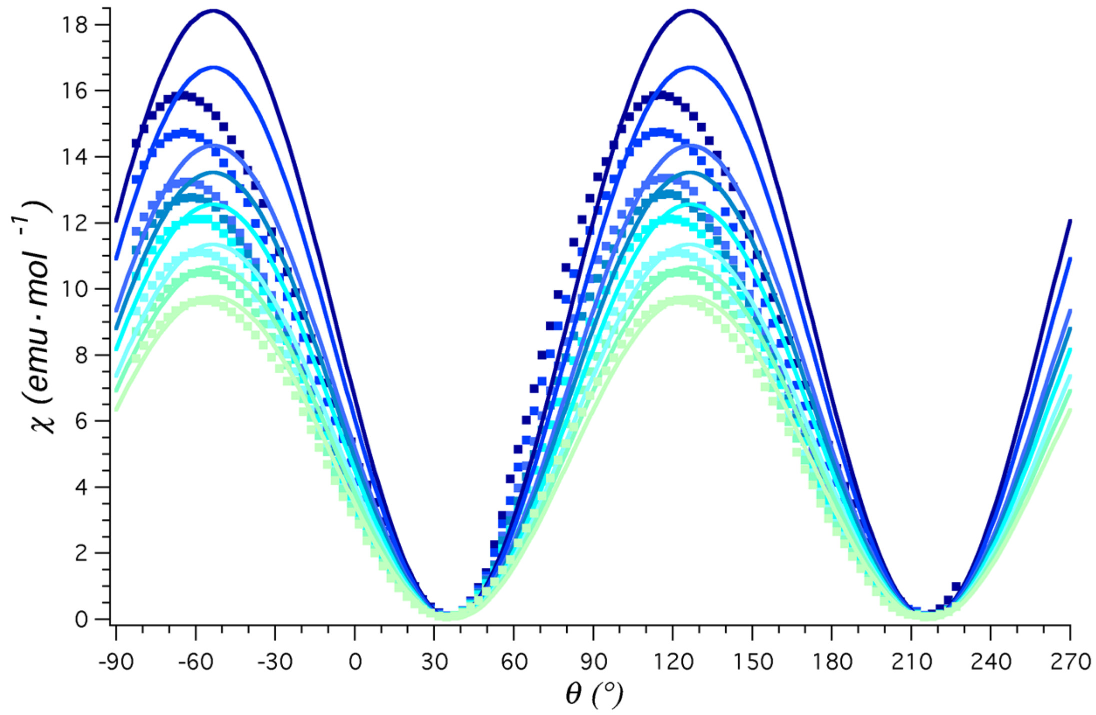

Fig. S3.7: Angular dependence of the magnetic susceptibility at different temperatures for Rot X at H = 1 kOe. From top to bottom: 1.8, 2, 2.35, 2.5, 2.7, 3, 3.2 and 3.5 K. Experiment (solid circles); REC model (solid lines).

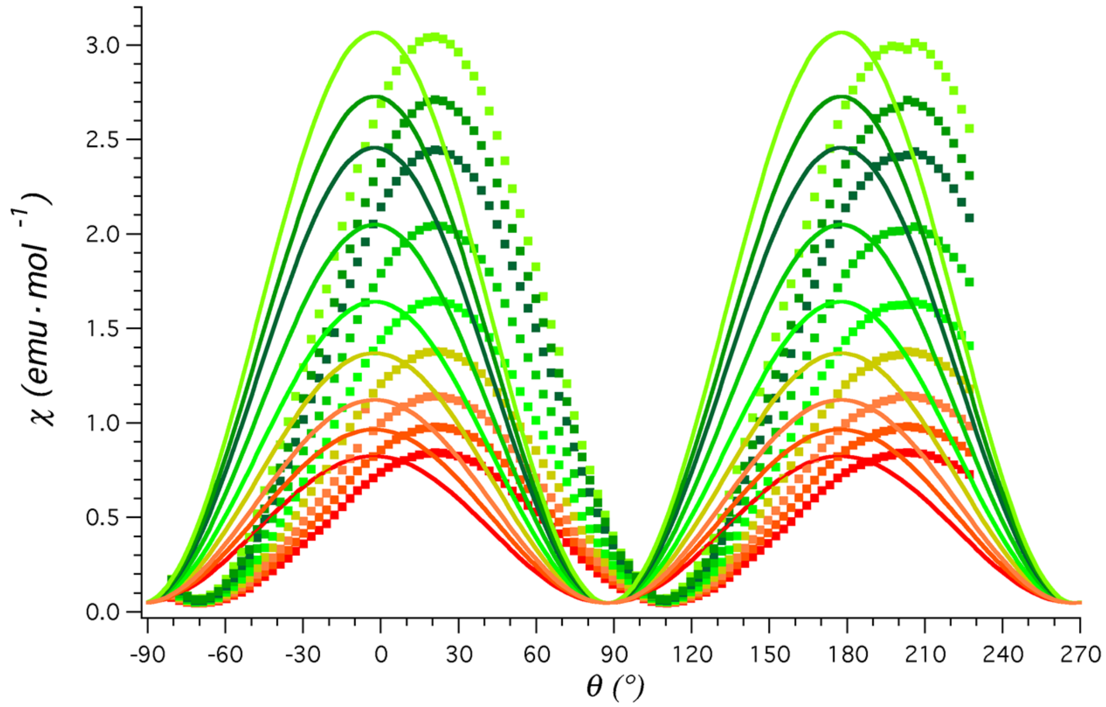

Fig. S3.8: Angular dependence of the magnetic susceptibility at different temperatures for Rot Y at H = 1 kOe. From top to bottom: 4, 4.5, 5, 6, 7.5, 9, 11, 12.8 and 15 K. Experiment (solid circles); REC model (solid lines).

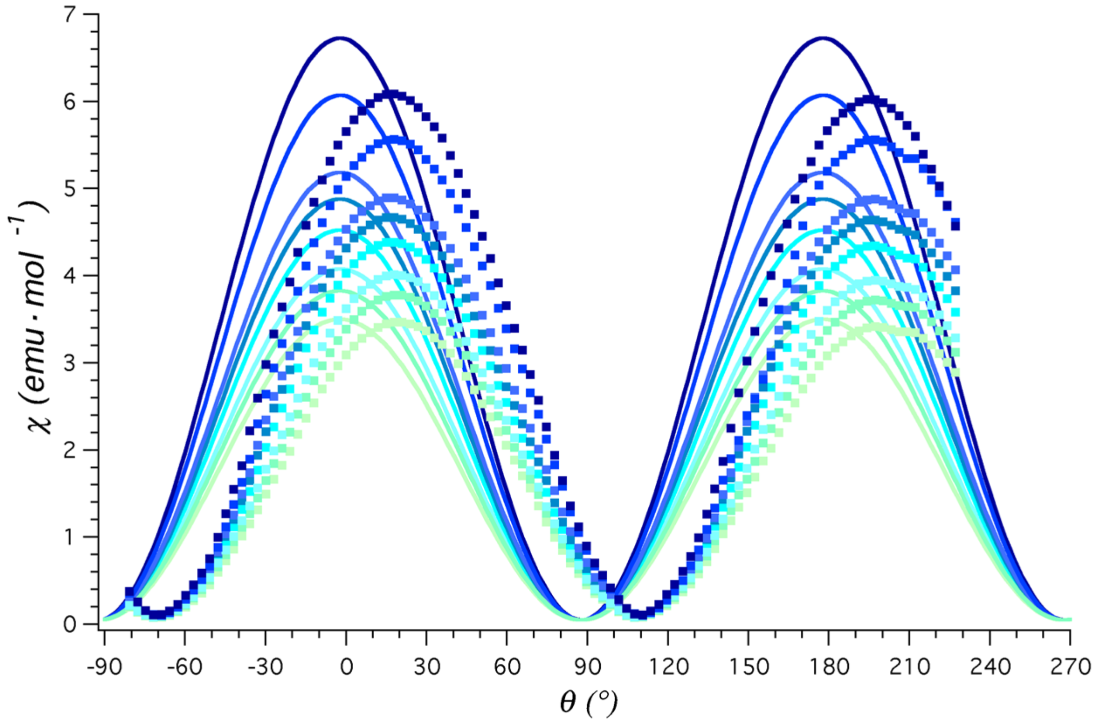

Fig. S3.9: Angular dependence of the magnetic susceptibility at different temperatures for Rot Y at H = 1 kOe. From top to bottom: 1.8, 2, 2.35, 2.5, 2.7, 3, 3.2 and 3.5 K. Experiment (solid circles); REC model (solid lines).

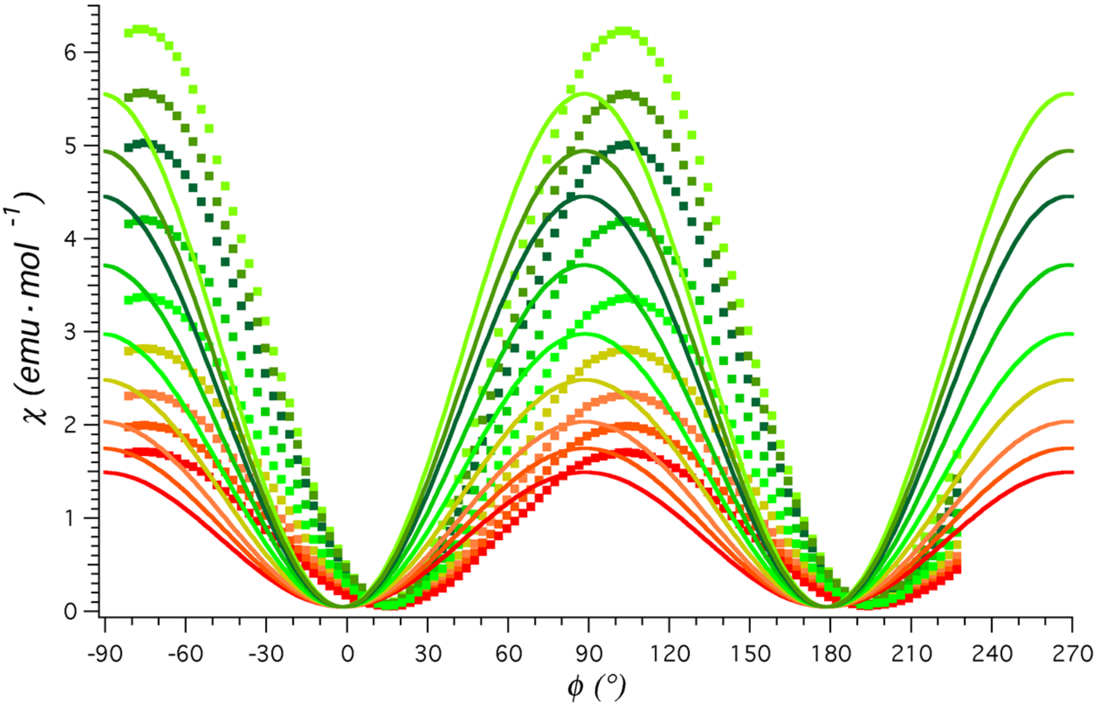

Fig. S3.10: Angular dependence of the magnetic susceptibility at different temperatures for Rot Z at H = 1 kOe. From top to bottom: 4, 4.5, 5, 6, 7.5, 9, 11, 12.8 and 15 K. Experiment (solid circles); REC model (solid lines).

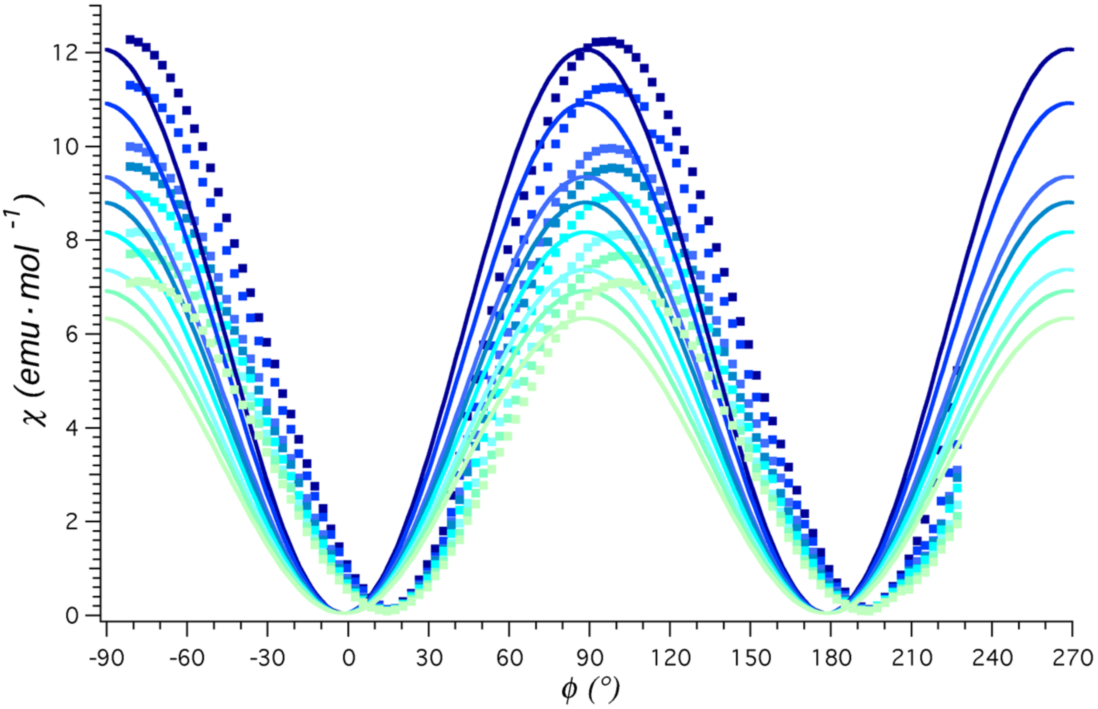

Fig. S3.11: Angular dependence of the magnetic susceptibility at different temperatures for Rot Z at H = 1 kOe. From top to bottom: 1.8, 2, 2.35, 2.5, 2.7, 3, 3.2 and 3.5 K. Experiment (solid circles); REC model (solid lines).

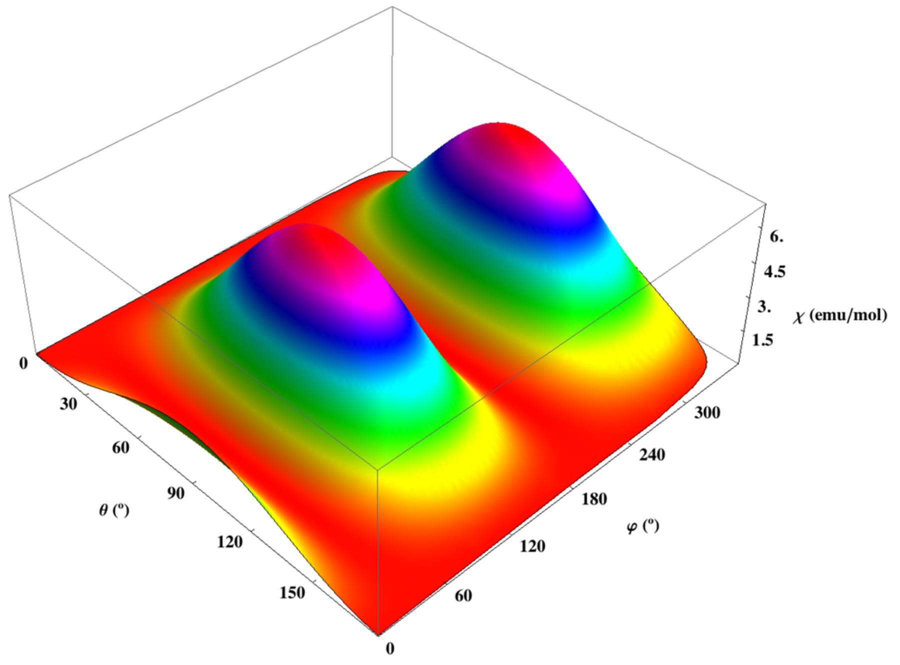

Fig. S3.12: Cylindrical map projection of the calculated susceptibility angular dependence at 5 K and H = 1 kOe corresponding to Rot X.

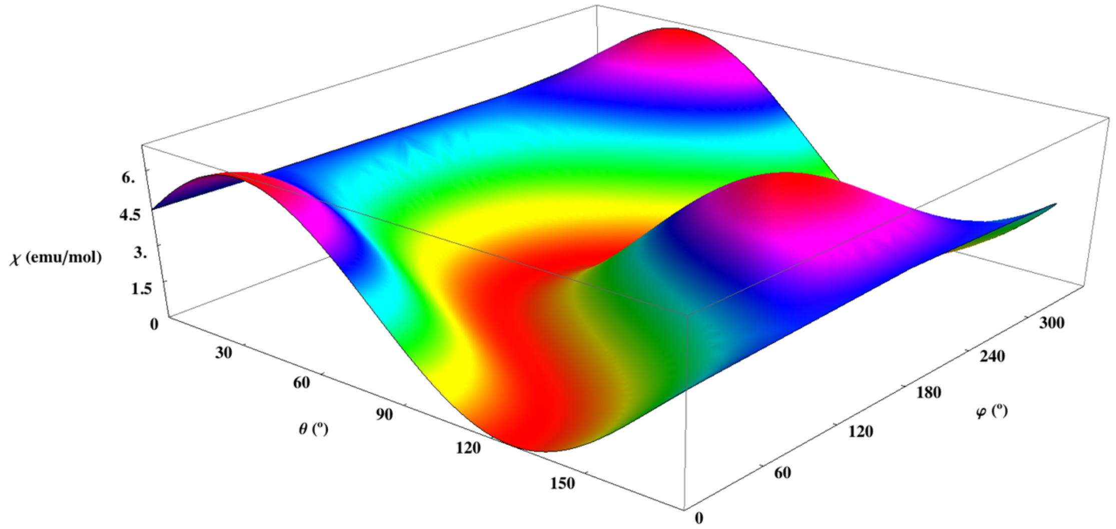

Fig. S3.13: Cylindrical map projection of the calculated susceptibility angular dependence at 5 K and H = 1 kOe corresponding to Rot Y.

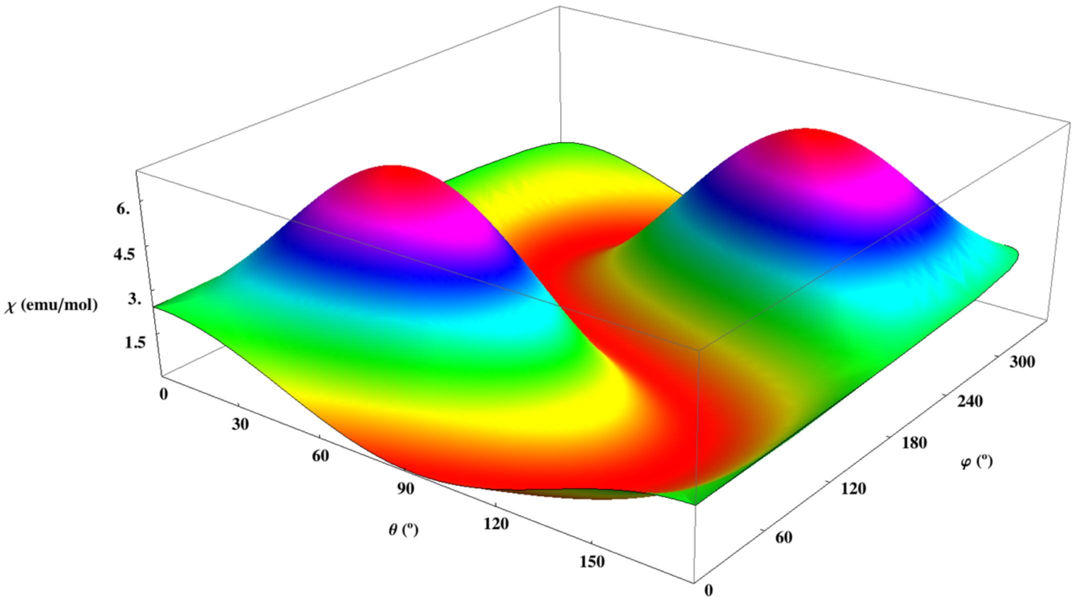

Fig. S3.14: Cylindrical map projection of the calculated susceptibility angular dependence at 5 K and H = 1 kOe corresponding to Rot Z.

SI4 Prediction of the properties of five related β-diketonate complexes

The REC parameters[1] determined in the present work for the ^{t}Bu-acac ligand (D_r = 0.57 Å and Z_i = 0.677) and the ones obtained for the bpy ligand in *Chem. Sci.*, 2013, 4, 938-946 (D_r = 1.25 Å and Z_i = 0.133) are extrapolated to a series of Dy^{III} and Er^{III} compounds with similar ligands. Thus, the magnetic and spectroscopic properties of the related β-diketonate compounds: $Dy(acac)_3(phen)$,[2] $Dy(acac)_3(dpq)$, $Dy(acac)_3(dppz)$,[3] $Er(h)_3(bipy)$ and $Er(h)_3(bath)$,[4] where acac = acetylacetonate, phen = 1,10-phenanthroline, dpq = dipyrido[3,2-f:2',3'-h]-quinoxaline, dppz = dipyrido[3,2-a:2',3'-c]-phenazine, h = 2,4-hexanedione and bath = bathophenanthroline, are predicted using the REC model in the SIMPRE computational package. Of course, in all these five cases, the donor atoms of the theoretically threated ligands are not exactly equivalent to the ones parameterized, thus we have to understand this results as an approximation. However, the calculated wave functions with this method indicate clearly the possibility of obtaining SMM behaviour as demonstrated experimentally. The main contributions to the wave function in the easy axis are that of $M_J = \pm 15/2$, especially larger in the three studied Dy^{III} derivatives (Table S4.1). The values of the first excited state calculated by CASSCF (supporting information of *Nature Comm.*, 2013, 4, 2551) are comparable to the ones reported herein as well as the easy axis direction. The predicted temperature-dependent magnetic susceptibility shows a good agreement with experimental data in the five complexes. Note that temperature-independent paramagnetism and a correction factor F to correct possible deviations between the reported and real weight of the sample measured have been added.

Table S4.1: First excited state of the ground multiplet calculated by CASSCF and the REC model in the present work of five related β-diketonate complexes. % M_J contribution to the ground doublet calculated by the REC model.

	CASSCF (cm^{-1})	REC (cm^{-1})	Amplitude of $\|M_J\rangle$ contributing to ground doublets (REC)
$Dy(acac)_3(phen)$	142	151	80% $\|\pm15/2\rangle$ + 17% $\|\pm11/2\rangle$
$Dy(acac)_3(dpq)$	133	150	78% $\|\pm15/2\rangle$ + 17% $\|\pm11/2\rangle$
$Dy(acac)_3(dppz)$	160	180	81% $\|\pm15/2\rangle$ + 17% $\|\pm11/2\rangle$
$Er(h)_3(bpy)$	-	46	58% $\|\pm15/2\rangle$ + 7% $\|\pm13/2\rangle$ +18% $\|\pm9/2\rangle$ + 11% $\|\pm7/2\rangle$
$Er(h)_3(bath)$	-	57	75% $\|\pm15/2\rangle$ + 8% $\|\pm7/2\rangle$

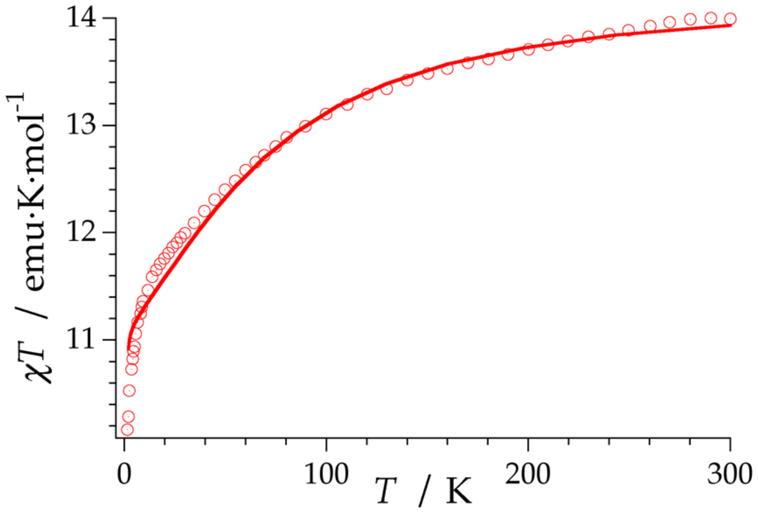

Fig. S4.1: χT product of Dy(acac)$_3$(phen) (red circles) from 2 to 300 K and prediction (solid line). TIP/diamagnetic correction = +0.0015; F = 0.92.

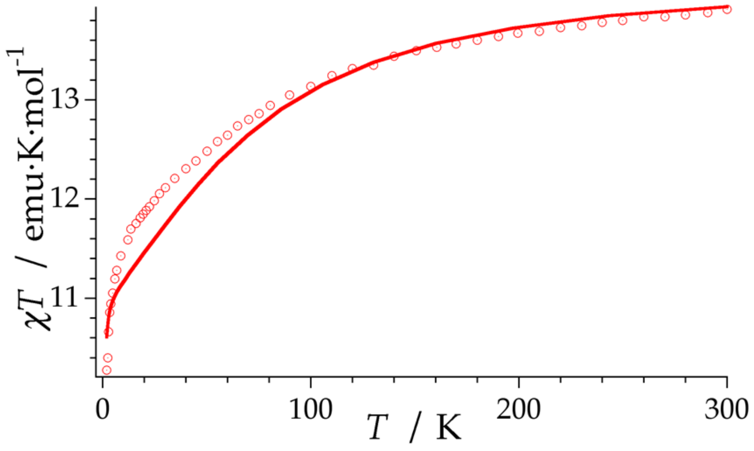

Fig. S4.2: χT product of Dy(acac)$_3$(dpq) (blue circles) from 2 to 300 K and prediction (solid line). TIP/diamagnetic correction = +0.002; F = 0.93.

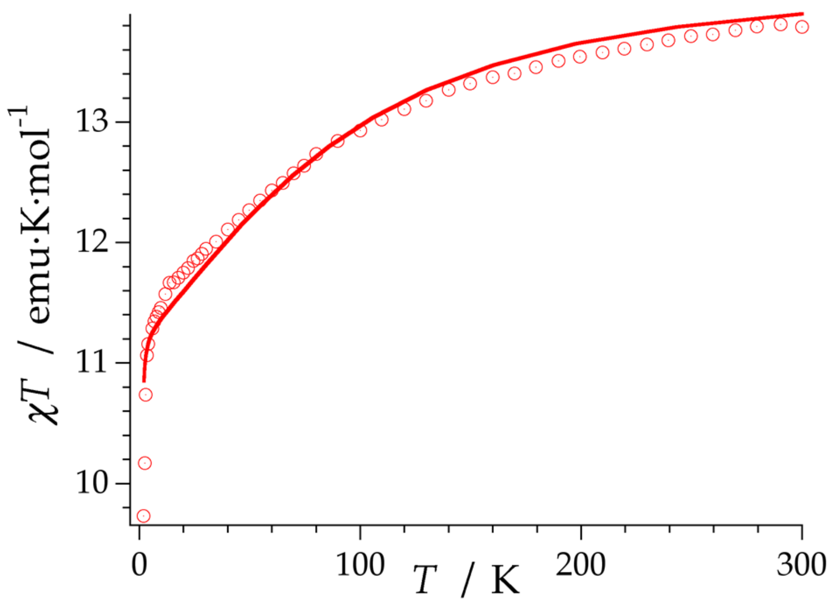

Fig. S4.3: χT product of Dy(acac)$_3$(dppz) (green circles) from 2 to 300 K and prediction (solid line). TIP/diamagnetic correction = +0.0016; F = 0.97.

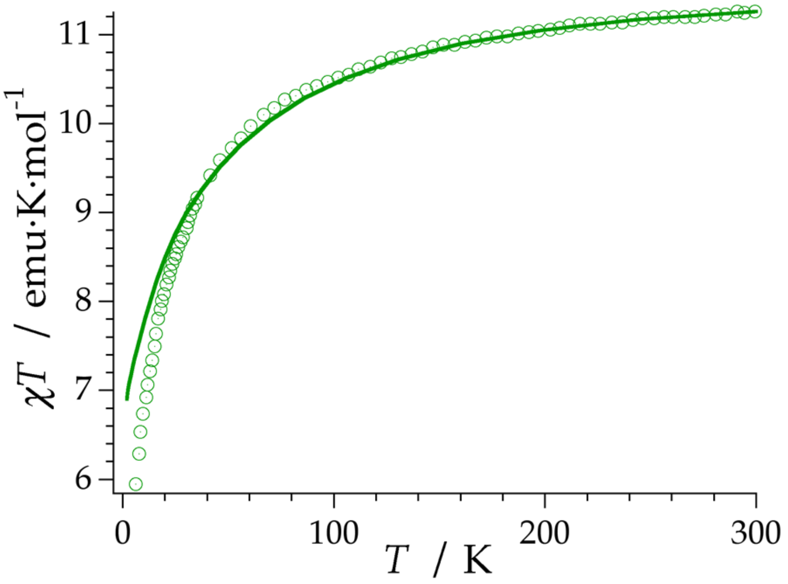

Fig. S4.4: χT product of Er(h)$_3$(bpy) (green circles) from 2 to 300 K and prediction (solid line). F = 0.93.

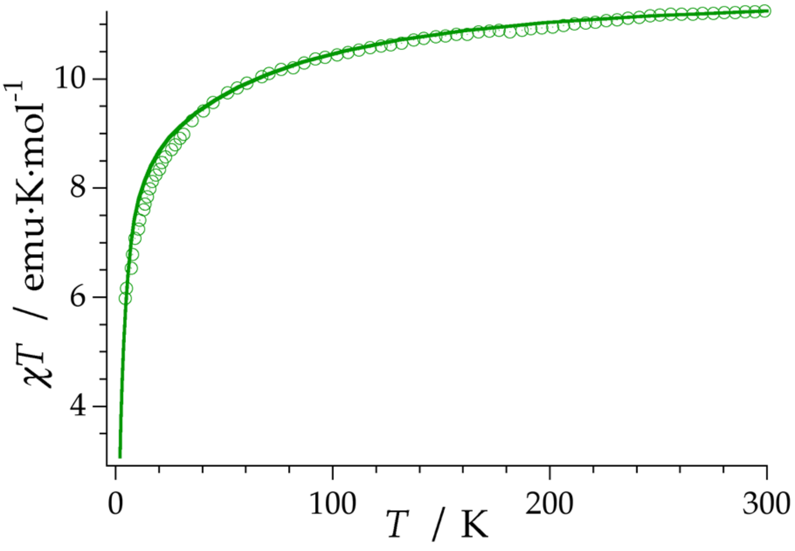

Fig. S4.5: χT product of Er(h)$_3$(bath) (green circles) from 2 to 300 K and prediction (solid line). F = 0.98.

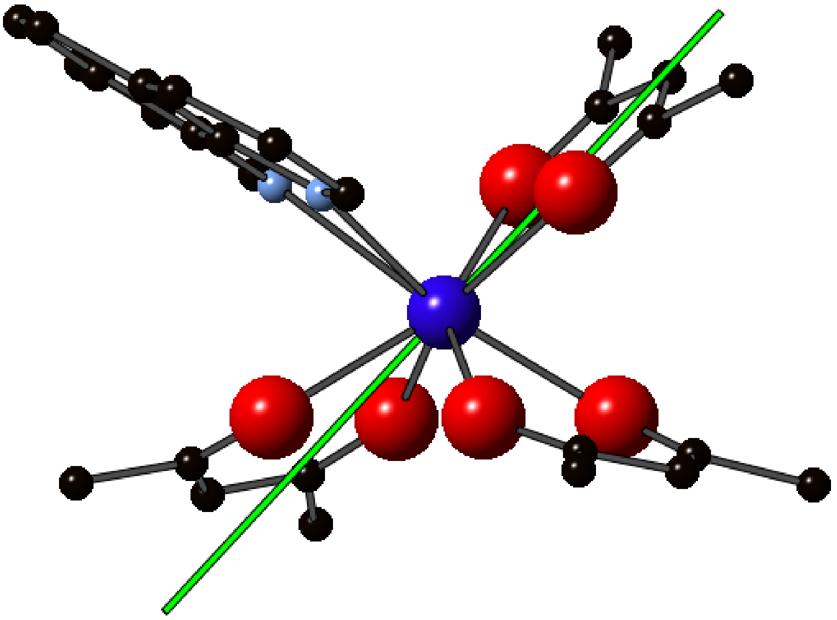

Fig. S4.6: Magnetic principal axis determined by the REC model in Dy(acac)3(phen).

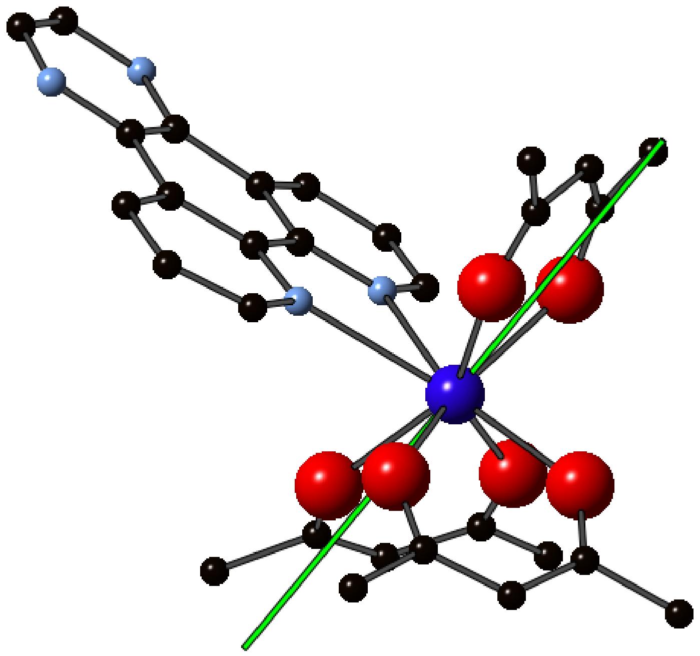

Fig. S4.7: Magnetic principal axis determined by the REC model in Dy(acac)3(dpq).

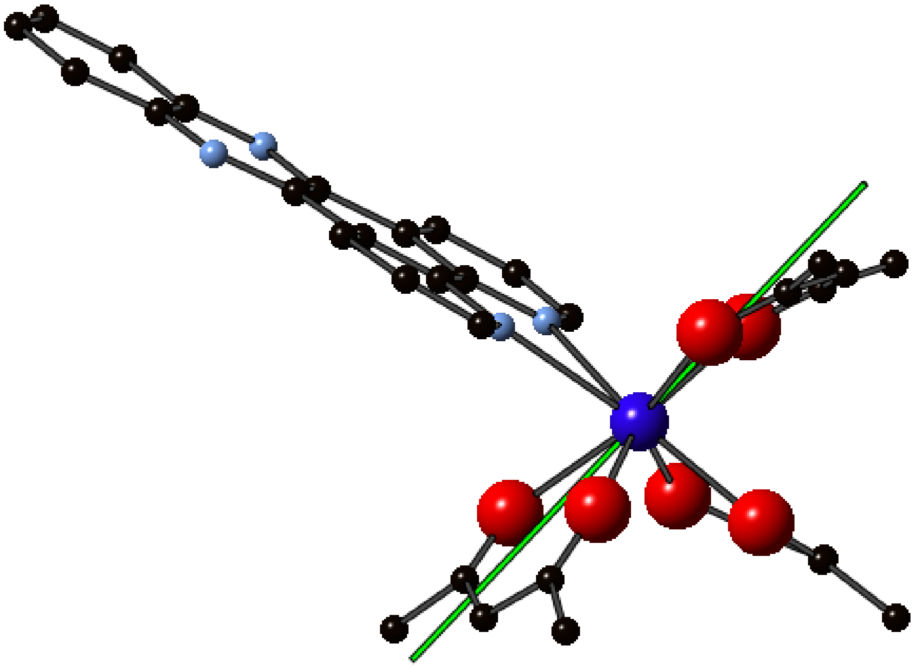

Fig. S4.8: Magnetic principal axis determined by the REC model in Dy(acac)3(dppz).

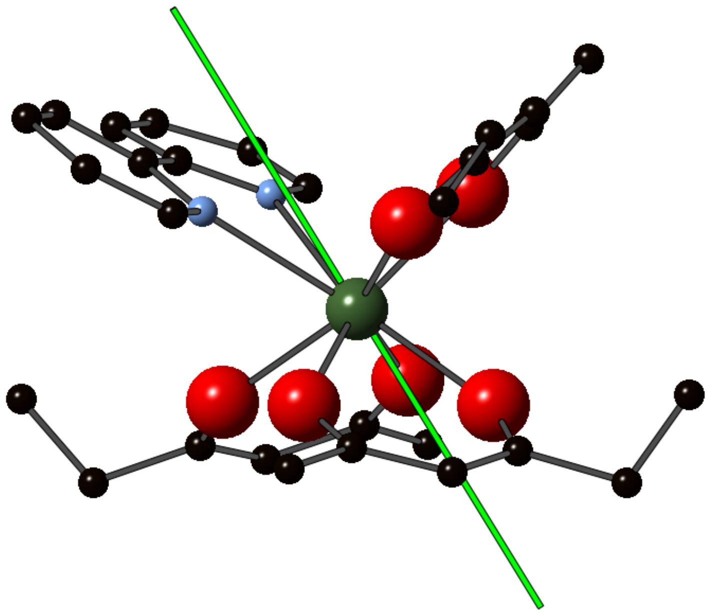

Fig. S4.9: Magnetic principal axis determined by the REC model in Er(h)3(bpy).

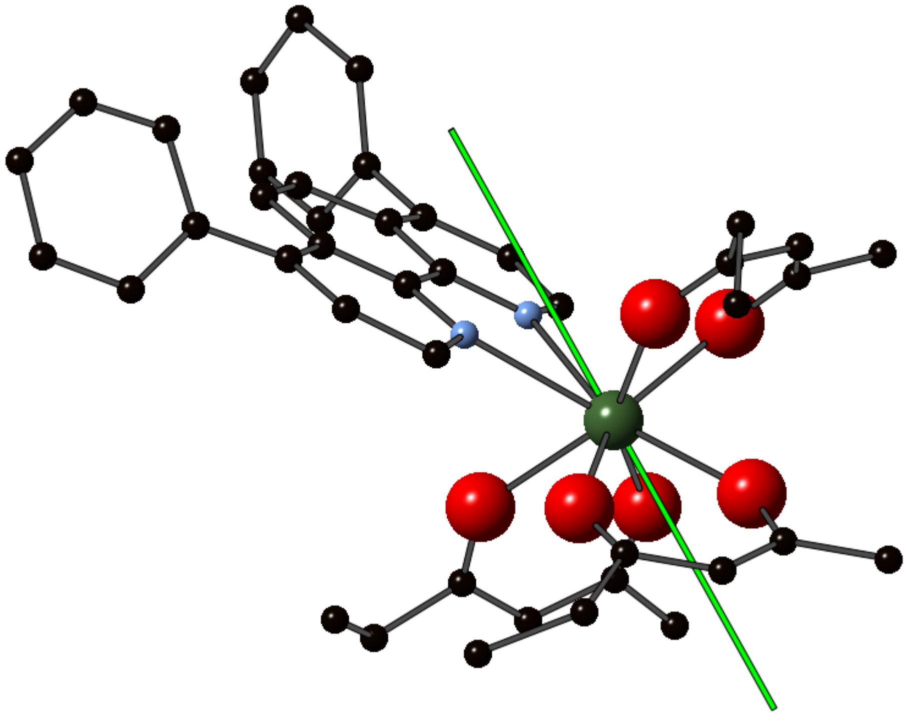

Fig. S4.10: Magnetic principal axis determined by the REC model in Er(h)3(bath).

---

[1] Baldoví, J.J.; Borras-Almenar, J.J.; Clemente-Juan, J.M.; Coronado, E.; Gaita-Ariño, A.; *Dalton Trans.*, **2012**, *41*, 13705−13710.

[2] Chen, G.-J.; Gao, C.-Y.; Tian, J.-L.; Tang, J.; Gu, W.; Liu, X.; Yan, S.-P; Liao, D.-Z.; Cheng, P., *Dalton Trans*. **2011**, *40*, 5579−5583

[3] Chen, G.-J.; Guo, Y.-N.; Tian, J.-L.; Tang, J.; Gu, W.; Liu, X.; Yan, S.-P; Cheng, P.; Liao, D.-Z., *Chem.Eur. J.* **2012**, *18*, 2484−2487.

[4] Ramos Silva, M; Martín-Ramos, P., Coutinho, J.T.; L.C.J. Pereira; Martín-Gil, J., *Dalton Trans*., **2014**, *43*, 6752-6761
